# Financial Toxicity in Selected Head and Neck Cancers: A Scoping Review of Measurement, Burden, and Outcomes

**DOI:** 10.3390/cancers18091378

**Published:** 2026-04-26

**Authors:** Madhuri Desai, Emanuel Fernandes Pinheiro, Ekta Pandey, Geetpriya Kaur, Neetu Sinha, Rui Amaral Mendes

**Affiliations:** 1 HEADS Doctoral Programme, Department of Community Medicine, Information and Health Decision Sciences (MEDCIDS), Faculty of Medicine, University of Porto, Rua Doutor Plácido da Costa, 4200-450 Porto, Portugal; up201800952@edu.med.up.pt; 2 RISE-Health, Department of Community Medicine, Information and Health Decision Sciences (MEDCIDS), Faculty of Medicine, University of Porto, Alameda Prof. Hernâni Monteiro, 4200-319 Porto, Portugal; epandey@med.up.pt (E.P.); up202314194@edu.med.up.pt (G.K.); up202401667@edu.med.up.pt (N.S.); 3 Faculty of Medicine, University of Porto, Alameda Professor Hernâni Monteiro, 4200-319 Porto, Portugal; up201905718@edu.med.up.pt; 4 PDICSS Doctoral Programme, Department of Community Medicine, Information and Health Decision Sciences (MEDCIDS), Faculty of Medicine, University of Porto, Rua Doutor Plácido da Costa, 4200-450 Porto, Portugal; 5 Department of Oral and Maxillofacial Medicine and Diagnostic Sciences, Case Western Reserve University, 10900 Euclid Ave., Cleveland, OH 44106-7342, USA

**Keywords:** financial toxicity, head and neck cancer, survivorship, rehabilitation, health equity, scoping review

## Abstract

**Background/Objectives**: Financial toxicity (FT) is increasingly recognised as a critical dimension of the cancer care continuum, reflecting both objective financial burden and subjective financial distress arising from cancer-related care. Head and neck cancers (HNC) may be particularly vulnerable to FT because treatment often involves multimodal care, functional morbidity, prolonged rehabilitation, and disruption to employment. This scoping review mapped and synthesised the literature on FT in a focused subset of head and neck cancers (HNC), namely malignancies of the oral cavity, oropharynx, nasopharynx, sinonasal tract, and major and minor salivary glands. **Methods**: A scoping review was conducted in accordance with the methodological guidance of the Joanna Briggs Institute for scoping reviews to identify and synthesise studies addressing FT in the selected HNC subsites. Searches were undertaken in MEDLINE, Embase, Scopus, Web of Science, CINAHL, EconLit, and Global Index Medicus for English-language studies published between 1 January 2015 and 1 January 2025. The search window was restricted to this period to capture the more contemporary evolution of FT as a distinct research construct in oncology. Eligible studies included adult patients and reported patient-level FT outcomes, including direct costs, indirect costs, out-of-pocket expenditure, financial hardship, financial distress, employment disruption, or related economic strain. Findings were synthesised narratively and organised thematically. **Results**: Twenty-five studies published between 2015 and 2025 were included. The evidence base was dominated by cross-sectional and retrospective designs, with limited prospective follow-up and very little intervention-focused research. FT was conceptualised heterogeneously across studies, spanning direct expenditure, indirect and non-medical costs, subjective financial distress, and coping-related consequences. Questionnaire-based approaches were used in 13 studies, but only a smaller subset employed FT-specific instruments such as COST. Across the literature, FT was most commonly associated with lower income, weaker financial protection, employment disruption, rural residence in some settings, and more intensive treatment. Reported downstream associations included poorer quality of life, psychological distress, care alteration, and work-related burden, although evidence for treatment delay or survival effects was more limited and should be interpreted cautiously. **Conclusions**: In this focused HNC subset, FT appears multidimensional, socially patterned, and clinically relevant. However, the literature remains methodologically fragmented, with inconsistent measurement and sparse longitudinal evidence. Future work should prioritise validated and tumour-specific assessment strategies, prospective study designs, and evaluation of mitigation interventions that address both direct and indirect burden across the cancer continuum.

## Simple Summary

Financial toxicity is increasingly recognised as an important consequence of cancer care, but its manifestations are not uniform across tumour groups. In selected head and neck cancers, the burden may be especially complex because treatment commonly generates prolonged functional morbidity, rehabilitation needs, employment disruption, and indirect household costs. This scoping review maps the available literature on financial toxicity in cancers of the oral cavity, oropharynx, nasopharynx, sinonasal tract, and major and minor salivary glands. This is particularly important because treatment in these cancers often entails prolonged rehabilitation, recurrent indirect costs, and disruption to employment, all of which may sustain financial burden beyond active treatment. Across 25 included studies, the evidence suggests that financial toxicity is multidimensional, socially patterned, and associated with poorer quality of life, distress, and work-related burden. At the same time, the literature remains methodologically heterogeneous, with limited longitudinal evidence and inconsistent use of validated financial toxicity measures. The review supports a more tumour-sensitive operational approach to financial toxicity assessment in this field and highlights clear priorities for future research, survivorship planning, and equity-oriented clinical care.

## 1. Introduction

Financial toxicity (FT) has emerged as an important dimension of the cancer care continuum, reflecting both the objective financial burden associated with treatment-related expenditure and the subjective financial distress experienced by patients and households. It is increasingly understood as a multidimensional construct that may include direct medical costs, direct non-medical costs, income loss, debt, depletion of savings, altered care-seeking, and psychosocial strain [1,2]. Although FT has been widely examined across oncology, its expression is not uniform across tumour groups, healthcare systems, or phases of survivorship. Recent HNC-focused reviews and primary studies have also highlighted the relevance of out-of-pocket costs and broader patient-borne economic burden in this field [3,4,5].

Head and Neck cancers (HNC) comprise a heterogeneous group of malignancies affecting anatomically and functionally critical sites, often requiring intensive multimodal treatment with substantial consequences for speech, swallowing, nutrition, appearance, and social participation. Selected head and neck cancers are especially relevant in this regard. The subsites addressed in this review frequently require aggressive and prolonged multimodal treatment, often followed by functional sequelae affecting swallowing, speech, dentition, appearance, and work ability. These clinical trajectories may generate not only high treatment-related expenditure, but also substantial rehabilitation needs, indirect costs, and survivorship-related financial burdens [5,6,7].

FT in this clinical setting should therefore not be reduced to a narrow accounting of direct treatment charges. Out-of-pocket expenditure related to supportive care, transport for specialised treatment, assistive devices, and ongoing rehabilitation may be substantial, particularly when combined with lost income or diminished work capacity. Emerging evidence from the primary studies included here suggests that FT is associated with poorer health-related quality of life, greater psychological strain, caregiver burden, and potentially maladaptive coping responses, although the strength and causal direction of these relationships remain incompletely defined [8,9,10,11,12,13,14].

A further challenge is methodological. FT is measured in heterogeneous ways and with differing levels of validation. Instruments such as the COmprehensive Score for Financial Toxicity (COST), the EORTC QLQ-C30 financial difficulty item, author-developed hardship surveys, administrative cost analyses, and other economic indicators have all been used. This heterogeneity complicates comparison across studies and weakens confidence in claims about prevalence, severity, and downstream effects. The distinction between FT-specific instruments, broader quality-of-life tools containing financial items, and purely economic indicators also needs to be handled more carefully than is often the case in the literature [4,12,15,16,17,18,19,20,21].

Despite these burdens, the FT literature in HNC remains fragmented, particularly in relation to study design, FT measurement approaches, patient populations, healthcare-system contexts, and outcome reporting, with limited synthesis across different health systems, sociodemographic strata, and phases of the cancer journey. For the purposes of this review, a focused definition of HNC was adopted, restricted to malignancies of the oral cavity, oropharynx, nasopharynx, sinonasal tract, and the major or minor salivary glands. These tumour groups share complex treatment pathways, substantial functional morbidity, prolonged rehabilitation needs, and high direct and indirect costs, making them particularly susceptible to FT.

By contrast, laryngeal, hypopharyngeal, tracheal, thyroid, and cutaneous head and neck cancers were excluded because their clinical trajectories, treatment algorithms, survivorship patterns, and cost structures differ sufficiently to introduce conceptual heterogeneity and reduce interpretability. The objectives of this scoping review were therefore to map and synthesise the existing literature on FT in this defined HNC subset, summarise the principal drivers of objective burden and subjective distress, and chart the reported impact of FT on clinical outcomes, treatment adherence, and health-related quality of life. More specifically, the review aimed to clarify how FT has been defined and measured in this selected HNC subset, identify the principal economic domains and vulnerability factors described, and examine the reported associations between FT and patient, clinical, and survivorship-related outcomes.

## 2. Materials and Methods

### 2.1. Scoping Review Framework

This scoping review was conducted to map the extent, range, and nature of the available literature on FT in the selected HNC subsites and to identify the main concepts, evidence gaps, and study types represented in the field. The review was undertaken in accordance with contemporary Joanna Briggs Institute guidance for scoping reviews and was reported in line with PRISMA-ScR principles [22,23]. This scoping review was registered retrospectively on the Open Science Framework (OSF) on 30 March 2026 (https://osf.io/bwsef/overview), thus providing a time-stamped record of the review methods and scope.

### 2.2. Search Strategy and Data Sources

A comprehensive search strategy was developed and refined with supervisory input. Searches were conducted in MEDLINE (via PubMed), Embase, Scopus, Web of Science, CINAHL, EconLit, and Global Index Medicus. Search terms combined population-focused concepts related to malignancies of the oral cavity, oropharynx, nasopharynx, sinonasal tract, and major or minor salivary glands with FT-related terms, including financial toxicity, financial burden, financial hardship, financial distress, economic burden, catastrophic health expenditure, out-of-pocket expenditure, productivity loss, income loss, employment disruption, and return to work. The search was restricted to English-language studies published between 1 January 2015 and 1 January 2025. This time window was selected to capture the more contemporary evolution of FT as a distinct research construct and to capture recent evidence generated after the wider operationalisation of FT in oncology literature.

The PubMed strategy combined Medical Subject Headings and title/abstract terms for the relevant HNC subsites with FT-related terms using Boolean operators. The strategy was then adapted for each database to maximise sensitivity while preserving relevance. The full PubMed search strategy is provided in Supplementary Material S1.

Studies were eligible if they included adults aged 18 years or older with malignancies of the oral cavity, oropharynx, nasopharynx, sinonasal tract, or the major or minor salivary glands, and reported any aspect of FT at patient level, including direct costs, indirect costs, financial hardship, financial burden, financial distress, or related economic strain. These subsites were selected because they share a substantial burden of multimodal treatment, functional morbidity, rehabilitation needs, and survivorship-related cost implications, thereby preserving greater clinical and economic coherence within the review.

Quantitative, qualitative, and mixed-methods studies were eligible, and systematic reviews were screened for reference harvesting but not included in the final synthesis.

### 2.3. Eligibility Criteria

Studies were excluded if they focused primarily on laryngeal, hypopharyngeal, thyroid, tracheal, or cutaneous head and neck cancers; did not report FT-related outcomes; involved paediatric populations; or did not provide an English-language full text. Editorials, commentaries, and other papers without empirical or substantive data were also excluded from the synthesis. The review was restricted to English-language studies because of feasibility constraints and the absence of resources for formal translation; this may have introduced language bias and is acknowledged as a limitation.

### 2.4. Study Selection

All citations were imported into Covidence systematic review software (https://www.covidence.org/, Veritas Health Innovation, Melbourne, Australia) and screened in two stages: title/abstract review followed by full-text assessment. After de-duplication, 1040 records entered screening. Four reviewers, working initially in two pairs, assessed eligibility at each stage. Disagreements were resolved through discussion and conflict resolution until consensus was reached. The final review included 25 studies. Screening was conducted independently by reviewer pairs at both stages. Prior to full screening, calibration was undertaken on a subset of records to improve consistency in applying the eligibility criteria. Disagreements were resolved through discussion and, where necessary, adjudication by a senior reviewer until consensus was reached.

### 2.5. Data Extraction and Charting

Data extraction and charting were undertaken after full-text review using a Covidence-based template that was subsequently expanded for the purposes of this review. In addition to study identifiers, authorship, year, design, sample size, and setting, the extraction framework captured cancer subsite, disease stage where reported, treatment characteristics, insurance or financial protection variables, and more granular patient-level financial information, including direct and indirect costs, out-of-pocket expenditure, catastrophic expenditure, employment disruption, return-to-work outcomes, and other indicators relevant to FT. Data extraction was undertaken using a structured Covidence-based template. Initial data extraction was undertaken by one reviewer and checked by a second reviewer, with discrepancies resolved through discussion and consensus.

### 2.6. Approach to Evidence Synthesis

Findings were synthesised narratively and organised according to the thematic structure of the review: study characteristics; conceptualisation and measurement of FT; economic burden and cost reporting; clinical and treatment-related drivers; sociodemographic and structural determinants; geographic and health-system variation; clinical, psychosocial, and survivorship-related outcomes; mitigation strategies; and equity-related considerations. No formal critical appraisal was undertaken, as the purpose of this scoping review was to map the breadth and characteristics of the evidence base rather than to generate pooled estimates or formal quality-weighted conclusions.

## 3. Results

### 3.1. Search Results

**Figure 1:**
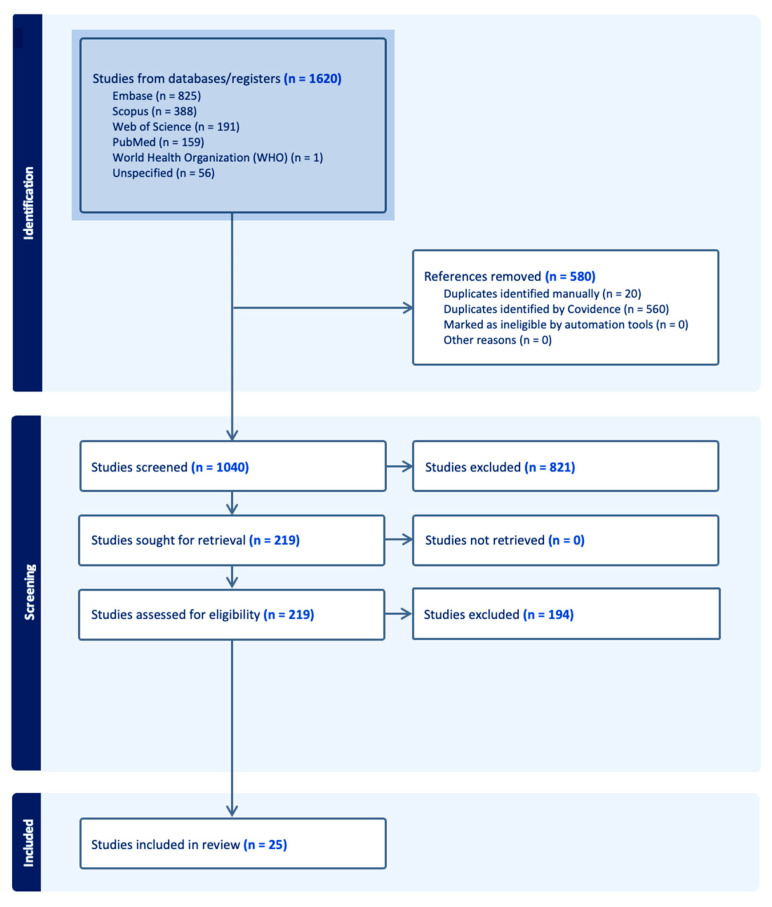
PRISMA flow diagram outlining the study selection process.

The literature search identified 1620 records across databases and registers. After removal of 580 duplicates, 1040 records were screened by title and abstract. A total of 219 full-text articles were assessed for eligibility, of which 194 were excluded. Twenty-five studies were ultimately included in the scoping review (Figure 1).

The most frequent reasons for exclusion at full-text stage were the absence of direct patient-level FT measurement, studies focused on health-system or hospital costs rather than individual financial burden, wrong outcomes, inappropriate publication type, wrong patient population, mixed cancer populations without separate HNC data, non-English language, and subsites outside the scope of the review.

### 3.2. Study Characteristics

Twenty-five studies published between 2015 and 2025 met the inclusion criteria. The included studies were conducted across a broad range of settings, including the United States, Germany, India, China, Malaysia, Canada, Sweden, Australia, and Sri Lanka, indicating that FT in HNC has been examined across diverse healthcare systems and socioeconomic contexts. Study characteristics are summarised in Table 1. Larger administrative datasets and economic analyses tended to provide broader estimates of healthcare-system and survivorship burden, whereas smaller cross-sectional studies more often captured patient-reported financial strain, quality-of-life effects, and work-related consequences.

### 3.3. Conceptualisation and Measurement of FT in HNC

Across the included studies, FT was conceptualised in heterogeneous ways, spanning objective financial burden, subjective financial distress, and, in some cases, behavioural responses under economic strain. Measurement approaches are summarised in Table 2. Thirteen studies used questionnaire-based approaches drawing on validated components, including COST and the EORTC QLQ-C30 financial difficulty item; however, only a smaller subset used FT-specific instruments rather than broader quality-of-life measures with a financial domain. Other studies relied on economic cost analyses, out-of-pocket expenditure metrics, or author-defined hardship surveys. This methodological heterogeneity remains one of the central limitations of the literature [12,15,16,17,18,19]. Importantly, validated questionnaire components did not always equate to FT-specific measurement, as some studies relied on broader quality-of-life instruments that included only a financial domain rather than a dedicated FT construct.

### 3.4. Domains of Financial Toxicity

The economic dimensions captured across studies are summarised in Table 3. FT in HNC extended well beyond direct treatment charges. Direct medical costs were frequently reported, but direct non-medical costs and indirect costs were also recurrent, including transport, accommodation, food, productivity loss, reduced work participation, and withdrawal from employment. Out-of-pocket expenditure was inconsistently reported, and studies varied substantially in whether they adopted a healthcare-system, patient, household, or societal perspective [6,9,11,25,26,28,34,35]. Direct medical costs did not necessarily equate to out-of-pocket expenditure, as several studies reported healthcare-system or insurer-level costs rather than costs directly borne by patients.

These findings reinforce that FT in HNC cannot be reduced to treatment billing alone. For many patients, the burden appeared to be distributed across travel, lodging, household expenditure, wage loss, and the financial consequences of treatment-related functional impairment.

### 3.5. Clinical and Treatment-Specific Drivers of FT

Clinical factors were frequently associated with greater FT. Several studies suggested that more advanced disease and the need for multimodal treatment increased the likelihood of financial burden, which is clinically plausible given the longer treatment duration, greater toxicity, and more extensive rehabilitation requirements likely to follow. Treatment intensity therefore appeared to function less as a simple exposure than as a gradient of economic vulnerability, although the largely cross-sectional nature of the evidence limits stronger causal inference [10,11,12,25].

### 3.6. Sociodemographic and Economic Determinants

Lower income emerged as the most consistent determinant of FT across the included studies. Other recurrent vulnerability factors included incomplete insurance or financial protection, employment disruption, productivity loss, rural residence, and minority status in some healthcare contexts. Taken together, these findings suggest that FT in selected HNCs is not merely an incidental economic after-effect of treatment, but is strongly shaped by prior socioeconomic position and the extent of protection available against illness-related costs [9,10,12,26,35].

### 3.7. Geographic and Health-System Variation

The sources and expression of FT varied across health systems. In higher-income settings with broader public coverage, such as Germany, Sweden, and Canada, FT more often appeared to arise through income loss, rehabilitation expenditure, and other indirect burdens rather than treatment charges alone. In middle-income settings, including Malaysia, India, Sri Lanka, and China, direct medical costs, catastrophic expenditure, and productivity loss were more prominent [6,17,25,28,30,31,34,35]. FT seems to be present across systems, but its composition varies according to the architecture of healthcare financing and social protection.

### 3.8. Impact of FT on Clinical and Psychosocial Outcomes

Clinical, psychosocial, employment, and survivorship-related outcomes associated with FT are summarised in Table 4. Seventeen studies reported financial burden, eight reported quality-of-life impact, six psychological distress, six care alteration, three treatment delay, five healthcare utilisation effects, eight employment impact, three return-to-work outcomes, and six survival associations. Reported downstream associations most commonly involved poorer quality of life, employment impact, psychological distress, and care alteration, while evidence relating FT to treatment delay, return to work, or survival was less frequent and should be interpreted cautiously given the limited number of studies and differences in design [10,12,13,14,17,18].

To provide an overall view of the reported outcomes, the frequency of each outcome across studies is summarised below (Table 5).

Taken together, these findings indicate that FT in selected HNCs is more than a descriptive economic observation. It appears to be associated with important implications for wellbeing, care experience, and survivorship, although stronger longitudinal and interventional evidence is still needed before firmer causal conclusions can be drawn [10,12,13,14,17,18].

### 3.9. Survivorship, Rehabilitation, and Return to Work

FT in HNC extended beyond active treatment and into survivorship. Ongoing rehabilitation needs, including speech and swallowing therapy, dental rehabilitation, and nutritional support, contributed to the long-tail cost of care. The return-to-work literature was limited, but the available studies suggested that although many patients did resume employment, persistent fatigue, dysphagia, speech impairment, and related functional limitations often hindered work ability and delayed occupational reintegration [7,11,12,31,32].

### 3.10. Interventions and Mitigation Strategies

Evidence on mitigation strategies was limited. The included literature remained predominantly descriptive rather than interventional, and few studies directly evaluated financial navigation, counselling, transport assistance, or structured survivorship support as FT-reduction strategies. Nonetheless, the available evidence suggests that earlier financial counselling, better access to rehabilitation, support with travel and accommodation, and vocational or return-to-work support may represent relevant avenues for mitigation [9,12]. This interpretation is consistent with broader oncology reviews, which suggest that financial navigation, direct assistance, insurance and cost-decision support, and structured counselling are among the most promising current intervention categories, albeit within a still heterogeneous evidence base [36,37].

### 3.11. Equity and Social Determinants of Health

The findings support a clear equity framing. Lower-income households consistently showed greater vulnerability to catastrophic expenditure, while rural patients often faced greater travel burden and minority groups in some settings experienced disproportionate financial strain. FT in selected HNCs should therefore be understood not only as an economic consequence of cancer care, but also as a mechanism through which pre-existing social disadvantage may be amplified [9,12,35].

## 4. Discussion

This scoping review demonstrates that FT in selected HNCs is multidimensional, persistent, and socially stratified. Across the 25 included studies, FT was not confined to direct medical expenditure, but extended to transport, accommodation, lost income, reduced work capacity, and long-term rehabilitation needs. In this respect, these tumour groups exemplify a form of cancer-related financial burden that is both intensive and prolonged, often continuing well beyond active treatment [10,11,12,31,32].

The review also highlights major methodological weaknesses. The evidence base is dominated by cross-sectional and retrospective designs, and the measurement of FT remains conceptually inconsistent. Some studies assessed objective expenditure, others subjective distress, and others a mixture of both, often without clearly distinguishing among them. Although thirteen studies used questionnaire-based approaches drawing on validated components, only a smaller subset employed FT-specific instruments, which limits comparability and weakens confidence in prevalence estimates. The lack of longitudinal data is especially problematic because FT in this field is likely to evolve across active treatment, early survivorship, and longer-term rehabilitation. This concern is fully in keeping with broader oncology measurement literature, which also highlights the need for multidimensional and psychometrically robust FT measures [2,20,21].

This raises the question of whether selected HNCs warrant a distinct FT framework. On balance, the answer is yes, though not in the sense of an entirely separate conceptual model. The general architecture of FT remains applicable, encompassing objective burden, subjective distress, and coping consequences [1,2]. However, these tumour groups appear to require a more tailored operational framework within that broader model, one that explicitly incorporates rehabilitation, functional morbidity, employment disruption, caregiver effects, and survivorship-related expenditure. Generic FT instruments may still be useful, but they are unlikely on their own to capture the full economic experience of these patients [8,14,20,21].

The review further suggests that FT in selected HNCs should be understood as an equity issue. Lower income, rural geography, employment insecurity, and incomplete financial protection were repeatedly associated with greater vulnerability. This is especially important because the burden of these cancers often falls on populations that are already socially or economically disadvantaged. FT in this context is not simply an unfortunate by-product of treatment; it forms part of a broader pattern in which disease intersects with structural precarity [9,10,12,26,35].

Health-system variation also emerged as a major theme. In settings with broader public coverage, FT often arose through income loss, rehabilitation costs, and indirect burden rather than treatment charges per se. In settings with weaker financial protection, direct medical expenditure and catastrophic household cost remained prominent. This suggests that FT is a common endpoint reached through different pathways, and that mitigation strategies must be context-sensitive rather than universal. This pattern is also consistent with the broader oncology FT literature, in which financial burden is common across settings but its dominant drivers vary according to the structure of healthcare financing and social protection.

Clinically, these findings support the integration of FT assessment into selected HNC care pathways. Screening should be considered especially for patients with advanced disease, anticipated multimodal treatment, lower income, rural residence, or insecure employment. Policy-wise, the evidence suggests that interventions should not be limited to treatment coverage alone. In many settings, major burden lies in indirect costs, wage loss, transport, and rehabilitation. Financial protection in these cancers therefore requires a broader lens, linking oncology care with social support, work reintegration, and survivorship services. This point is reinforced by wider FT and return-to-work literature, which underscores the importance of multidimensional screening, supportive employment environments, and structured survivorship support [2,37,38].

### 4.1. Limitations

Despite clear patterns, several methodological and conceptual limitations within the primary literature must be acknowledged. First, substantial heterogeneity in FT definitions and measurement approaches limits cross-study comparability. Although several studies used validated questionnaire components, only a smaller subset employed FT-specific instruments such as COST, while many others relied on bespoke surveys, administrative datasets, or broader quality-of-life measures with a financial domain. Comparing absolute cost values across studies is also problematic because of differences in currency, inflation, time period, and health-system context.

Secondly, the evidence base is dominated by cross-sectional surveys and retrospective analyses, with only two studies adopting a longitudinal design. This limits the ability to understand how FT evolves across the cancer continuum and obscures whether burden peaks during active treatment, early survivorship, or later rehabilitation. Survey-based designs may also be vulnerable to selection bias if the most socioeconomically vulnerable patients are less likely to respond or remain in follow-up. In addition, caregiver burden and inter-subsite variation remain insufficiently captured in the current literature.

Finally, limitations inherent to this scoping review should be acknowledged. The exclusion of non-English literature may have introduced language bias, and grey literature or non-peer-reviewed organisational reports were not systematically searched. The focused subsite definition, while conceptually defensible, also means that the conclusions should not be generalised indiscriminately to all head and neck cancers.

### 4.2. Implications for Practice and Future Research

The identified gaps provide several priorities for future research and practice. To reduce methodological heterogeneity, future investigations should prioritise the development or selection of core, validated, and multidimensional FT measures that can be used consistently across studies while remaining sensitive to tumour-specific realities [20,21]. Because selected HNCs often affect populations already facing socioeconomic disadvantage, employment vulnerability, and weaker financial protection, FT should also be approached and studied explicitly as an issue of health equity.

In practical terms, FT screening could be incorporated into multidisciplinary HNC clinics, survivorship follow-up, and rehabilitation pathways, with referral to social work, financial counselling, or vocational support where appropriate. In geographically dispersed populations, transport support and telehealth may represent particularly relevant implementation targets.

Clinical practice and policy should also address the chronic survivorship-related financial burden observed in selected HNCs. The out-of-pocket costs associated with long-term dental, speech, and swallowing rehabilitation represent a distinct burden that may remain under-recognised and underinsured. Given the limited evidence on return-to-work outcomes, future research should incorporate vocational and occupational reintegration more explicitly, recognising the unique aesthetic, vocal, and functional barriers that survivors may face [38]. Finally, the literature contains very limited evaluation of mitigation strategies. Future interventional research should therefore examine the impact of financial counselling, transport and accommodation support, telehealth-enabled follow-up, and return-to-work interventions in this population [36,37].

## 5. Conclusions

FT in selected HNCs is a clinically relevant and socially patterned consequence of cancer care. It extends beyond direct treatment expenditure to encompass income loss, reduced work capacity, travel burden, and the prolonged costs of rehabilitation and survivorship. Although the current literature establishes the importance of the problem, it remains methodologically fragmented and limited by inconsistent conceptualisation and sparse longitudinal evidence. Future work should prioritise validated and multidimensional measurement, prospective tumour-sensitive designs, broader inclusion of under-represented settings, and integration of FT screening into routine care. The present findings also support greater incorporation of FT assessment into multidisciplinary HNC care, particularly in survivorship and rehabilitation settings. More broadly, this focused subset of HNCs may require a more tumour-sensitive operational framework for FT, given the distinctive combination of multimodal care, prolonged functional morbidity, and indirect household burden. Without this next step, FT in this field will remain well recognised yet incompletely understood, and opportunities to reduce inequity in care will continue to be missed.

## Figures and Tables

**Table 1:** Characteristics of Included Studies (n = 25).

Study	Country	Study Design	Sample Size	Cancer Type	Treatment Type	Stage Reported
Nocon 2021 [24]	USA	Economic evaluation	136	Mixed HNC	Multimodal	Not reported
Raman 2022 [25]	Malaysia	Mixed-methods	104	Oral cavity	Not reported	Early vs. late
Rast 2024 [19]	Germany	Prospective survey	200	HNC mixed	Multimodal	Mixed
Rast 2025 [17]	Germany	Cross-sectional survey	200	HNC mixed	Multimodal	Mixed
Edwards 2016 [26]	India	Cross-sectional survey	400	Oral cavity	Multimodal	Not reported
Lairson 2017 [27]	USA	Claims database analysis	467	Oropharyngeal	Surgery, RT, CT	Mixed
Massa 2018 [7]	USA	Cross-sectional survey	100	HNC survivors	Multimodal	Mixed
Yeoh 2020 [28]	Malaysia	Cross-sectional study	299	Nasopharyngeal	RT, CT	Mixed
Baddour 2021 [12]	USA	Cross-sectional survey	71	HNC survivors	Multimodal	Mixed
Jella 2021 [29]	USA	Cross-sectional survey	710	HNC survivors	Multimodal	Mixed
Zhou 2022 [30]	China	Cross-sectional survey	44,382	Nasopharyngeal	RT, CT	Mixed
Khan 2022 [9]	Canada	Longitudinal	657	HNC survivors	Multimodal	Mixed
Ehrsson 2023 [31]	Sweden	Cross-sectional survey	227	HNC survivors	Multimodal	Mixed
Martino 2017 [6]	Canada	Longitudinal	21	HNC survivors	Multimodal	Mixed
Morales 2020 [32]	Australia	Cross-sectional survey	68	HNC survivors	Multimodal	Mixed
Ma 2021 [10]	USA	Cross-sectional survey	284	HNC survivors	Multimodal	Mixed
Massa 2019 [33]	USA	Economic analysis	489	HNC survivors	Multimodal	Mixed
Thaduri 2023 [13]	India	Cross-sectional study	79	Oral cancer	Surgery, RT	Mixed
Amarasinghe 2019 [34]	Sri Lanka	Cross-sectional survey	69	Oral cavity	Surgery, RT	Mixed
Diao 2023 [18]	USA	Cross-sectional survey	396	HNC survivors	Multimodal	Mixed
Massa 2022 [11]	USA	Economic analysis	19,098	HNC survivors	Multimodal	Mixed
Thaduri 2025 [14]	India	Cross-sectional study	79	Oral cancer	Surgery, RT	Early vs. advanced
Luo 2025 [15]	China	Cross-sectional study	155	HNC survivors	Multimodal	Mixed
Jiang 2022 [16]	China	Cross-sectional study	210	HNC survivors	Multimodal	Mixed
Goswami 2023 [35]	India	Mixed-methods	100	Oral cavity	Multimodal	Mixed

Note: Study design labels reflect the primary analytic approach reported by the original authors; some categories are not mutually exclusive.

**Table 2:** Financial Toxicity Measurement Approaches.

Measurement Approach	Studies (n/%)	Example Studies
FT questionnaires/PROMs (COST, EORTC, FT surveys, distress scales)	16/64%	Baddour 2021 [12]; Luo 2025 [15]; Jiang 2022 [16]; Rast 2025 [17]
Direct medical cost analysis (cost-of-illness, expenditure studies)	10/40%	Lairson 2017 [27]; Goswami 2023 [35]; Yeoh 2020 [28]
Indirect costs/productivity loss (income loss, workdays lost)	11/44%	Khan 2022 [9]; Martino 2017 [6]; Thaduri 2023 [13]
Out-of-pocket (OOP) expenditure reporting	9/36%	Raman 2022 [25]; Baddour 2021 [12]; Massa 2022 [11]
Catastrophic health expenditure (CHE)	4/16%	Raman 2022 [25]; Goswami 2023 [35]
Economic indicators (OOP ratios, claims-based metrics)	5/20%	Zhou 2022 [30]; Nocon 2021 [24]; Massa 2019 [33]

Key insight: Several studies used validated questionnaire components, but only a smaller subset employed FT-specific instruments. Several studies reported multiple financial toxicity measurement approaches. Percentages are calculated based on the total number of included studies (n = 25).

**Table 3:** Cost Components and Economic Burden reported across studies.

Study	Direct Costs Reported	Indirect Costs	OOP Expenses	Cost Perspective
Nocon 2021 [24]	Yes	No	No	Healthcare system
Raman 2022 [25]	Yes	Yes	Yes	Household
Lairson 2017 [27]	Yes	No	Yes	Healthcare system
Yeoh 2020 [28]	Yes	Yes	No	Healthcare system
Khan 2022 [9]	No	Yes	Yes	Patient
Martino 2017 [6]	Yes	Yes	Yes	Societal
Amarasinghe 2019 [34]	Yes	Yes	Yes	Societal
Goswami 2023 [35]	Yes	Yes	Yes	Household
Massa 2019 [33]	Yes	No	No	Healthcare system
Massa 2022 [11]	Yes	No	Yes	Healthcare system

Note: Direct medical costs do not necessarily equate to out-of-pocket expenditure; some studies reported healthcare-system or insurer-level costs rather than costs directly borne by patients.

**Table 4:** Clinical and Psychosocial Outcomes Associated with Financial Toxicity.

Study	Financial Burden	QoL Impact	Psychological Distress	Care Alteration	Treatment Delay	Healthcare Utilization	Employment Impact	Return to Work	Survival Association
Nocon 2021 [24]	–	✓	–	–	–	–	–	–	–
Raman 2022 [25]	✓	✓	✓	✓	–	–	–	–	✓
Rast 2024 [19]	✓	✓	✓	–	✓	–	–	–	–
Rast 2025 [17]	✓	–	–	–	✓	–	✓	–	–
Edwards 2016 [26]	✓	–	✓	–	–	–	–	–	✓
Lairson 2017 [27]	–	✓	–	–	–	–	–	–	–
Massa 2018 [7]	✓	–	–	–	–	–	–	–	–
Yeoh 2020 [28]	✓	✓	✓	–	–	–	–	–	–
Baddour 2021 [12]	✓	✓	–	✓	–	–	✓	–	–
Jella 2021 [29]	–	–	–	✓	–	✓	✓	–	✓
Zhou 2022 [30]	–	✓	–	–	–	✓	–	–	–
Khan 2022 [9]	✓	✓	✓	–	–	–	–	✓	–
Ehrsson 2023 [31]	✓	–	–	–	–	–	–	✓	–
Martino 2017 [6]	✓	✓	✓	–	–	–	–	✓	–
Morales 2020 [32]	✓	–	–	–	–	–	–	✓	–
Ma 2021 [10]	–	–	–	–	–	–	–	–	✓
Massa 2019 [33]	✓	–	–	–	–	✓	–	–	✓
Thaduri 2023 [13]	✓	–	–	–	–	–	✓	–	–
Amarasinghe 2019 [34]	✓	✓	✓	–	–	–	–	–	–
Diao 2023 [18]	✓	–	–	✓	–	–	✓	–	✓
Massa 2022 [11]	–	✓	–	–	–	✓	–	–	✓
Thaduri 2025 [14]	✓	–	–	–	–	–	✓	–	–
Luo 2025 [15]	–	–	–	✓	–	–	–	–	✓
Jiang 2022 [16]	–	–	–	✓	✓	✓	✓	–	✓
Goswami 2023 [35]	✓	✓	✓	✓	–	✓	–	–	✓

Legend. ✓ = outcome reported. – = outcome not reported.

**Table 5:** Frequency of reported outcomes.

Outcome	Studies Reporting
Financial burden	17
QoL impact	8
Psychological distress	6
Care alteration	6
Treatment delay	3
Healthcare utilization	5
Employment impact	8
Return to work	3
Survival association	6

## Data Availability

All relevant information may be found in the OSF registration site already mentioned in the Methodology and further added in the Supplementary Material.

## Supplementary Materials

The following supporting information can be downloaded at: https://www.mdpi.com/article/10.3390/cancers18091378/s1. Supplementary Material S1. Full PubMed Search Strategy.
