# Supplementary material for: Financial Toxicity in Selected Head and Neck Cancers: A Scoping Review of Measurement, Burden, and Outcomes
Source: Cancers (Basel). 2026 Apr 26;18(9):1378. doi: 10.3390/cancers18091378 (PMC13163044; doi:10.3390/cancers18091378)
Supplement: Supplementary file 1 [file cancers-18-01378-s001.zip › Supplementary Material S1. Full PubMed Search Strategy.pdf]

Supplementary Material S1. Full PubMed Search Strategy

Search date range: 1 January 2015 to 1 January 2025
Database: MEDLINE (via PubMed)

This supplementary file provides the full PubMed search strategy used for the scoping review of financial toxicity in selected head and neck cancers. The search framework and review scope were also documented in the retrospectively registered OSF record ((https://osf.io/bwsef/overview).

The search strategy was developed in accordance with the review's Population–Concept–Context (PCC) framework. The population comprised adults diagnosed with malignancies of the oral cavity, oropharynx, nasopharynx, sinonasal tract, and the major or minor salivary glands. The concept was financial toxicity, defined to include both objective financial burden and subjective financial distress, including out-of-pocket costs, catastrophic expenditure, income loss, employment disruption, caregiver costs, and related material, psychological, and behavioural responses. The context included all geographical regions, health-system models, and phases of the cancer journey, including diagnosis, active treatment, rehabilitation, survivorship, return to work, and palliative care.

The PubMed search strategy combined MeSH terms and title/abstract terms for the selected head and neck cancer subsites with MeSH and title/abstract terms relating to financial toxicity and associated economic burden. The strategy was restricted to English-language studies published between 1 January 2015 and 1 January 2025. Syntax was adapted as necessary for the other databases searched in the main review.

Full PubMed search strategy

(
"Oral Neoplasms"[Mesh] OR
"Mouth Neoplasms"[Mesh] OR
"Oropharyngeal Neoplasms"[Mesh] OR
"Nasopharyngeal Neoplasms"[Mesh] OR
"Paranasal Sinus Neoplasms"[Mesh] OR
"Maxillary Neoplasms"[Mesh] OR
"Mandibular Neoplasms"[Mesh] OR
"Salivary Gland Neoplasms"[Mesh] OR
"oral cancer"[tiab] OR
"oral cavity cancer"[tiab] OR
"oropharyngeal cancer"[tiab] OR
"oropharynx cancer"[tiab] OR
"nasopharyngeal carcinoma"[tiab] OR
NPC[tiab] OR
"sinonasal cancer"[tiab] OR
"paranasal sinus cancer"[tiab] OR
"salivary gland cancer"[tiab] OR
"parotid cancer"[tiab] OR
"submandibular gland cancer"[tiab] OR
"sublingual gland cancer"[tiab] OR
"minor salivary gland"[tiab]
)
AND

(
“Financial Toxicity”[Mesh] OR
“Out-of-Pocket Costs”[Mesh] OR
“Treatment Costs”[Mesh] OR
“Financial Stress”[Mesh] OR
“Health Expenditures”[Mesh] OR
“Poverty”[Mesh] OR
“financial toxicity”[tiab] OR
“financial burden”[tiab] OR
“financial hardship”[tiab] OR
“financial distress”[tiab] OR
“economic burden”[tiab] OR
“economic hardship”[tiab] OR
“catastrophic health expenditure”[tiab] OR
“out-of-pocket”[tiab] OR
“medical bankruptcy”[tiab] OR
“cost of illness”[tiab] OR
“productivity loss”[tiab] OR
“income loss”[tiab] OR
“employment disruption”[tiab] OR
“indirect costs”[tiab] OR
“work disability”[tiab] OR
“return to work”[tiab]
)

This strategy was designed to balance sensitivity and relevance by combining a focused set of selected head and neck cancer subsites with a broad conceptual definition of financial toxicity and related economic consequences.
